# The Role of Regulatory T Cells in Epicutaneous Immunotherapy for Food Allergy

**DOI:** 10.3389/fimmu.2021.660974

**Published:** 2021-07-08

**Authors:** Guirong Liu, Manman Liu, Junjuan Wang, Yao Mou, Huilian Che

**Affiliations:** Key Laboratory of Precision Nutrition and Food Quality, Key Laboratory of Functional Dairy, Ministry of Education, College of Food Science and Nutritional Engineering, China Agricultural University, Beijing, China

**Keywords:** allergen-specific immunotherapy (AIT), epicutaneous immunotherapy (EPIT), food allergy, regulatory T cell (Treg cell), immune tolerance

## Abstract

In recent decades, a rapid increase in the prevalence of food allergies has led to extensive research on novel treatment strategies and their mechanisms. Mouse models have provided preliminary insights into the mechanism of epicutaneous immunotherapy (EPIT)-induced immune tolerance. In EPIT, antigen applied on the skin surface can be captured, processed, and presented in the lymph nodes (LNs) by Antigen-presenting cells (APCs). In the LNs, induction of regulatory T cells (Treg cells) requires both direct contact during antigen presentation and indirect mechanisms such as cytokines. Foxp3^+^CD62L^+^ Treg cells can exhibit the characteristics of hypomethylation of Foxp3 TSDR and Foxp3^-^LAP^+^ Treg cells, which increase the expression of surface tissue-specific homing molecules to exert further sustained systemic immune tolerance. Studies have shown that EPIT is a potential treatment for food allergies and can effectively induce immune tolerance, but its mechanism needs further exploration. Here, we review Treg cells’ role in immune tolerance induced by EPIT and provide a theoretical basis for future research directions, such as the mechanism of EPIT and the development of more effective EPIT treatments.

## Introduction

Food allergies are a growing concern given their increasing global incidence in recent decades. WHO has listed it as one of five major public health problems (1). Research shows that food allergies are more common in developed countries, and up to 8% of children and 5% of adults in Western countries suffer from food allergies (2, 3). Besides, the incidence of food allergies in children is higher than that of adults. Epidemiological surveys have shown that up to 4.5% to 13.5% of children in Japan suffer from food allergies (4).

Strictly avoiding allergenic foods after correct diagnosis and preparing for adrenaline injection in accidental exposure cases is still the most effective therapy at present. However, some common allergenic foods, such as milk and eggs, are commonly used as food additives in processed foods. In addition, food allergen information labeling is not perfect, and it is difficult to avoid altogether accidental food allergen consumption, which is challenging for patients and their families (5). To date, studies related to food allergies have mainly focused on allergen-specific immunotherapy (AIT), DNA vaccines, dietary supplements, Chinese herbal formulae, adjuvant-enhanced immunotherapy, and the introduction of allergenic foods in the early stages of life (6, 7). AIT is a potentially effective treatment for food allergies, and its effectiveness and safety have been confirmed to some extent (8, 9). However, the specific mechanism needs further research.

The ultimate goal of AIT treatment is to enable patients to tolerate allergens, and various types of immune cells play an important role in this process. Accumulating evidence indicates that AIT may act by modifying the patterns of cytokines produced by helper T cells (Th) (10). Researches of mice and humans have identified Th2 cytokines [interleukin (IL)-4, IL-13, and IL-5] as major contributors to allergic disease (11–13), while AIT can bias the immune response of allergic patients to Th1 type immune response. Majori et al. (10) found that AIT has a significant effect on increasing the interferon-γ (IFN-γ, Th1 cytokine)/IL-4 ratio in peripheral blood CD4^+^ T cells, which is consistent with the observations of Ohashi (14) and Varney (15). Besides, Hamid et al. (16) showed that IL-12 can effectively stimulate the proliferation of Th1 T lymphocytes in AIT treatment, and IL-12 may inhibit late-phase responses after successful immunotherapy. In addition, AIT induced antigen-specific suppressive activity in CD4^+^CD25^+^ T cells of allergic individuals, which is in line with the immunosuppressive effect observed in non-allergic individuals, was observed in Jutel’s research (17). More specifically, the deviated immune response was characterized by suppressed proliferative T cells and Th1 and Th2 cytokine responses, and increased IL-10 and TGF-β secretion by allergen-specific T cells (17). Additionally, IL-10-producing regulatory B cells (Breg cells) also showed a potent suppressive capacity on antigen-specific CD4^+^ T-cell activation (18). Breg cells were observed most abundantly within the initial weeks of immunotherapy and gradually returned to baseline as AIT progressed (19). However, regulatory T cells (Treg cells) were observed in the later stages of AIT, indicating the role of Treg cells in the induction of long-term immune tolerance by AIT (20). Moreover, the critical role of Treg cells in various types of AIT has been further confirmed in mouse models (21, 22). Here, we review Treg cells’ role in immune tolerance induced by epicutaneous immunotherapy (EPIT) and provide a theoretical basis for future research on the mechanisms of EPIT and the development of more effective EPIT treatments.

## Regulatory T Cells

In 1971, Gershon and Kondo (23) first discovered a subset of T cells pretreated with thymocytes that may have immunosuppressive effects and prevent otherwise ‘helpful’ T cells from mediating heir function. Subsequent studies had also confirmed the existence of such suppressor T cells (Ts cells) (24–32). However, the development of Ts cells suffered a major blow in the early 1980s (33). Biochemical and molecular experiments questioned the interpretation of earlier studies, and the term “suppressor T cell” almost disappeared within a few years (33–35). As Darwin said, science is to sort out the facts, so that from the common rules and conclusions. For about 30 years, scientists had not given up on the study of Ts cells, they had to cover up their data in the name of “down-regulation” or “infectious tolerance or anergy”. As Ronald described, like a phoenix, negative regulatory T cells rose from these ashes to a position of prominence in today’s immunological thinking over precisely the interval from the demise of Ts to the present (33). Fortunately, since the late 1990s, the interest in Ts cells was reborn. In 1995, Sakaguchi et al. (36) discovered a subset of thymus-derived CD4^+^ T cells that continuously express CD25, the receptor α chains of IL-2, which can protect thymectomized mice from autoimmunity and was later named Treg cells (37). Since then, substantial researches have explored the immunosuppressive effects of Treg cells and their mechanisms. Apart from protecting from autoimmunity, Treg cells also play a role in other pathological and physiological immune responses, such as allergy (38), tumor immunity (39, 40), transplantation (41, 42), and microbial immunity (43, 44), and can also be targeted to suppress or enhance the immune responses in clinical settings (45).

Different studies showed that the expression of the transcription factor Foxp3 faithfully identifies these naturally occurring Treg cells (46–49). Moreover, loss-of-function mutations of the *Foxp3* gene lead to poor development of CD4^+^CD25^+^ Treg cells (47, 48). These findings together led people to believe that Foxp3^+^ Treg cells represent a stable cell lineage. Subsequent studies began to use Foxp3 as a “specific” molecular marker for Treg cells to reveal the molecular and cellular mechanisms of Treg cell differentiation and function (50). However, *Foxp3* alone does not control all aspects of Treg biology and is not the initiating factor in Treg development. A fact made clear as CD25^+^Foxp3^-^ Treg precursors in the thymus are already fate committed to the Treg cells lineage despite their lack of Foxp3 expression (51–54). Importantly, induction and maintenance of Foxp3 expression are two separable processes regulated by distinct *cis*-regulatory elements within the *Foxp3* locus (55). Treg cell-specific demethylation region (TSDR), that is, the region where the cytosine-guanine dinucleotide (CpG) site in Treg cells is completely demethylated, has been shown to be required for heritable maintenance of the stable and high expression of Foxp3 in dividing Treg cells and play a key role in Treg cells’ inhibitory function (55, 56). Furthermore, TSDR was thought to identify the “real” human Treg cells (57). Besides, Treg cells also express surface molecules such as costimulatory molecules CD28 (58), chemokine receptors CCL27/28 (CCR10) (59), CCL20 (CCR6) (60), and CCL17/22 (CCR4) (61).

In the literature, Tregs are divided into subpopulations according to differentiation sites and the expression of well-known functional markers. For the first time in 2009, Battaglia et al. (62) divided human Treg cells into three subgroups according to the expression levels of CD25, CD45R, and Foxp3: CD25^++^CD45RA^+^ (Foxp3^lo^) resting Treg cells (rTreg cells), CD25^+++^CD45RA^-^ (Foxp3^hi^) activated Treg cells (aTreg cells), and CD25^++^CD45RA^-^ (Foxp3^lo^) cytokine-secreting T cells. Of these, rTreg cells represent naive Treg cells, and aTreg cells represent effector Treg cells at different differentiation stages. The former two subgroups exert immunosuppressive functions, while cytokine-secreting T cells lack inhibitory activity (62). Despite the improvement in Treg cells biology, there are no specific markers to characterize human Treg cells, and the expression of Treg cell surface molecules is not constant. This classification still has certain limitations. Also, they can be further characterized by the site of differentiation: thymus-derived Treg cells (tTreg cells), peripherally induced Treg cells (pTreg cells), and *in vitro* induced Treg cells (iTreg cells) (63). tTreg cells usually include rTreg cells and aTreg cells (64). pTreg/iTreg cells are generated from conventional Foxp3^-^CD4^+^T cells, and the expression of Foxp3 can be induced after IL-2, retinoic acid, and TGF-β activates CD3 signaling molecules (63). However, a phenotypic distinction between tTreg cells and pTreg cells has not yet been established (65). Accumulating evidence indicates that tTreg cells and pTreg cells play different roles in different tissues. tTreg cells persist in the periphery, play a stable function role in maintaining dominant self-tolerance (45). Besides, due to the nature of pTreg cells differentiation (non-self-antigens and a particular TCR signaling combined with other signals), these cells are assumed to be more functional for maintaining mucosal tolerance (66–68).

**Figure 1:**
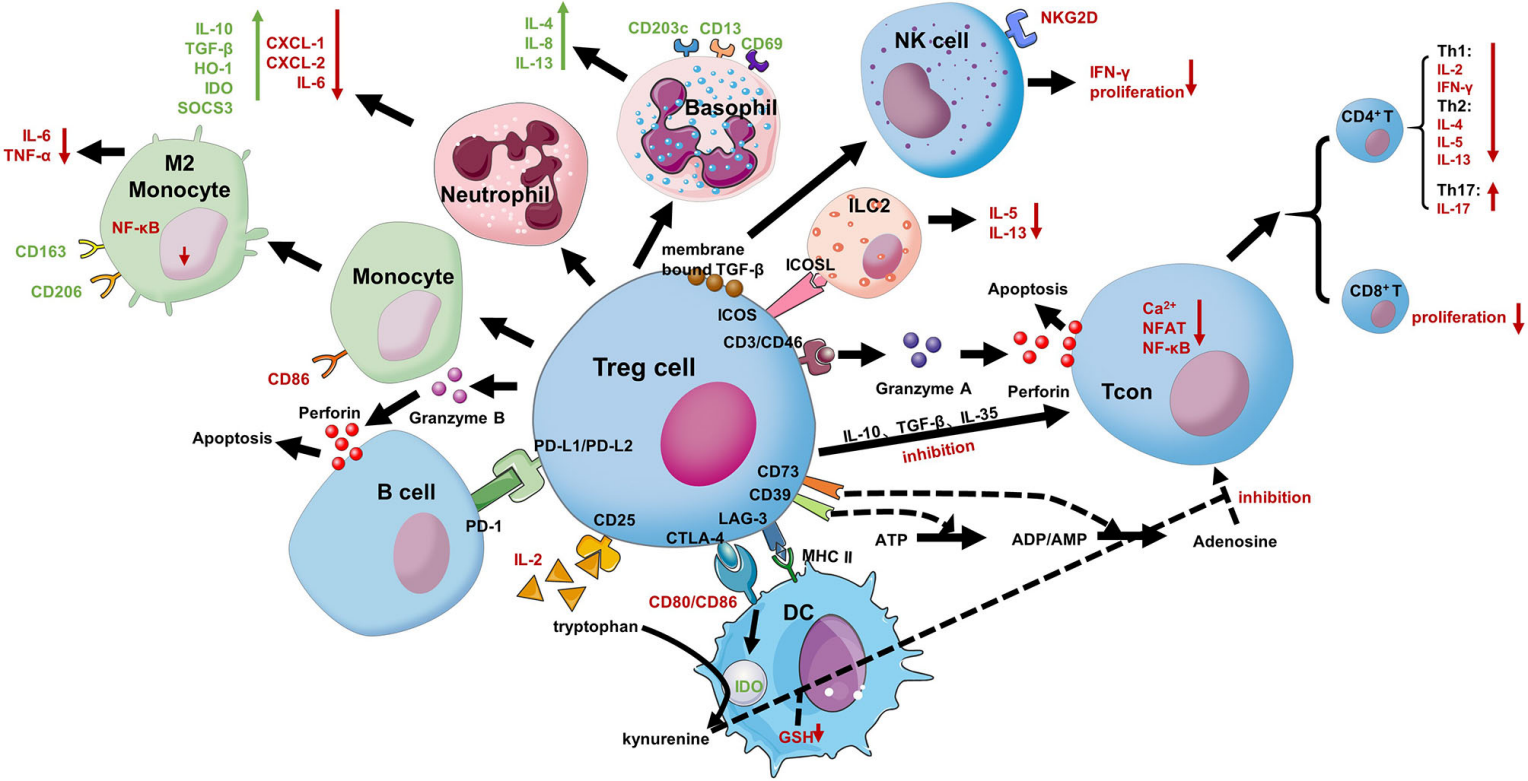
Treg cells suppressive mechanisms. Treg cells inhibit the proliferation and effects of NKs through membrane-bound TGF-β, mainly including inhibiting the expression of the latter’s surface protein NKG20 and the production of IFN-γ. Treg cells can inhibit the secretion of IL-5 and IL-13 by ILC2 in an ICOS/ICOSL-dependent manner, thereby inhibiting its function. Treg cells can inhibit conventional T cells (Tcons) action in many ways. 1) Treg cells can produce anti-inflammatory cytokines (IL-10, IL-35, and TGFβ) affecting Tcons; 2) they can release perforin and granzyme, which damage the target cell membrane leading to apoptosis; 3) Treg cells can also sequester, by the high expression of CD25, IL-2 from the microenvironment reducing effector Tcons proliferation; 4) Treg cells can quickly inhibit TCR-induced Ca^2+^, NFAT, and NF-κB signaling; 5) indirectly inhibiting Tcons by reducing the expression of CD80/CD86 on DCs through CTLA-4 (inhibit DC antigen presentation function) or disrupting the microenvironment in the immunological synapse provided by DCs (essential for T cell proliferation); 6) The expression of CD39 on Treg cells mediates the conversion of ATP to adenosine and reduces the proliferation of Tcons. Treg cells can directly affect B cells *via* PDL1/PD-1 interaction and DCs *via* CTLA-4 and LAG-3. CTLA-4 blocks co-stimulation, reducing CD80/CD86 expression, and it induces upregulation of IDO. Treg cells can inhibit B cells action and release granzyme B and perforin through the PD-1 signaling pathway to kill B cells. Treg cells can also bias monocytes to M2 macrophages, enhancing CD163 and CD206 on their surface molecules. They can similarly induce the suppressive phenotype of neutrophils and basophils and reduce the secretion of ILC2 cytokines.

Studies have shown that Treg cells’ level in children with food allergies is significantly downregulated, leading to a decline in immunosuppressive function. This decline can promote and aggravate allergies, which illustrates the critical role of Treg cells in maintaining immune tolerance in the body (69). As shown in **Figure 1**, Treg cells can directly exert immunosuppressive functions in several ways (67, 68): 1) secretion and production of IL-10, TGF-β, IL-35, as well as granzyme and perforin, which directly affect the proliferation, activation, and apoptosis of conventional T cells (Tcons) (70). Treg cells can inhibit the proliferation of Th1 and Th2 cells and the secretion of cytokines (such as Th1 cytokines: IL-2, IFN-γ; Th2 cytokines: IL-4, IL-5, and IL-13), they also can promote Th17 cells proliferate and secrete IL-17 to exert an inhibitory effect (68). At the same time the induction of high affinity effector and memory CD8^+^ T cells is reduced (67); 2)Treg cells can affect the proliferation, activation and apoptosis of B cells in the manner described in 1); 3) inhibition of TCR-induced Ca^2+^, NFAT, and NF-κB signaling in Tcons, and inhibition of B cells through the PDL1/PD-1 signaling pathway (51); 4) direct inhibition of the proliferation and effect of NK through membrane-bound TGF-β, mainly through inhibition of the expression of the latter’s surface protein NKG20 and the production of IFN-γ (71, 72); 5) inhibition of IL-5 and IL-13 secretion by ILC2s in an ICOS/ICOSL-dependent manner, thereby inhibiting its function (73); 6) direct inhibition of DCs through both the CTLA-4/CD80 and LAG-3/MHC II signaling pathways (68, 74); 7) acting on monocytes and granulocytes, inhibiting their cytokine secretion, differentiation, and antigen-presenting function (75, 76). Besides, Treg cells can act indirectly by 1) highly express CD25 to create a microenvironment lacking IL-2 so that Treg cells can “starve” surrounding cells that need this cytokine (77); 2) promoting extracellular ATP conversion into adenosine and AMP, which have immunosuppressive effects, by expressing CD39/CD73 (70); 3) indirectly inhibiting Tcons by reducing the expression of CD80/CD86 on DCs through CTLA-4 (inhibit DC antigen presentation function) or disrupting the microenvironment in the immunological synapse provided by DCs (essential for T cell proliferation) (67, 78).

## Allergen-Specific Immunotherapy for Food Allergy

AIT is considered the only treatment for allergic diseases that can effectively change the disease’s course. Its efficacy for allergic asthma, rhinitis, and allergic diseases has been confirmed (79–81). In recent years, AIT has been used to treat food allergies and is considered a potentially effective treatment for allergic diseases (82–84). The principle of AIT is to gradually increase the allergen dose to reduce the patient’s responsiveness to allergenic foods and ultimately achieve the goal of desensitization and sustained unresponsiveness (85).

AIT includes subcutaneous immunotherapy (SCIT), oral immunotherapy (OIT), sublingual immunotherapy (SLIT), and EPIT. Researches on SCIT for food allergies have shown that it can cause severe side effects, so it is generally not considered a treatment (86, 87). OIT is currently the most widely studied method for the treatment of food allergies. Studies have shown that OIT can effectively treat egg (88), milk (89, 90), and peanut (91, 92) allergies. It is worth noting that the world’s first approved food allergy treatment drug, PALFORZIA, was approved by the US Food and Drug Administration (FDA) to treat peanut allergy patients on January 31, 2020. Although clinical studies of PALFORZIA have shown that this oral immunotherapy can lead to rapid desensitization to peanut protein and improve allergy sufferers and their guardians’ quality of life, almost all participants reported adverse events (93). In addition, Chu et al. (94) systematically evaluated the potential risks of peanut OIT treatment. The results showed that, comparing with avoiding peanuts, the risk of allergic reactions during peanut OIT treatment increased 3.12 fold, and the risk of using epinephrine increased 2.21 fold. SLIT is used as a potential alternative to OIT. SLIT involves the administration of small drops of allergen extract (micrograms to milligrams) under the tongue for approximately 2 minutes, which is then eventually spit or swallowed (95, 96). During this process, it is absorbed by Langerhans cells (LCs) and is finally brought into the draining lymph nodes (dLNs) to induce antigen-specific tolerance (97). The dosage of SLIT is 1/100-1/1000 of OIT. The secondary effects of SLIT are mainly itching and oropharyngeal irritation. Although most studies have reported systemic adverse reactions, they are not common compared with OIT (98, 99). However, its effectiveness needs to be further explored.

## Epicutaneous Immunotherapy

In addition to injection or oral administration, the skin is also a promising treatment site for diseases. As a target area for treatment, skin has many advantages. First, as a non-vascularized tissue, the epidermis can strictly restrict the entry of allergens into the bloodstream, minimizing the risk of acute side effects. Second, skin is rich in antigen-presenting cells (APCs), especially immunomodulatory macrophages (100). Therefore, allergens can be transported in the intact skin, and by activating APCs, it can further promote the production of allergen-specific Treg cells, which can prevent and treat food allergies (101, 102). Third, compared with the oral route, applying the drug to the skin can prevent the substance from being chemically or enzymatically disintegrated in the gastrointestinal tract or liver, which is especially important for protein or peptide drugs that are widely used in immunotherapy, such as insulin (103) and antibody (104). Last but not the least, skin is a more accessible treatment site to manage, which provides convenience for patients to treat themselves at home. These advantages have aroused people’s interest in its development. In addition, as a skin application method, transdermal patches can be traced back to ancient China (around 2000 BC), when people began to apply medicated plasters containing multiple herbal ingredients to the skin as a treatment method (105, 106). With trial, error, clinical observation and evidence-based studies, transdermal patches are now widely used as cosmetic, topical and transdermal delivery systems (105). The studies of dermal application induced suppression were inspired by earlier observations showing that epicutaneous application of protein antigen on the skin in a form of a gauze dressing induces the synthesis of IL-4 and IL-13, which may potentially inhibit the immune response mediated by CD4^+^ Th1 lymphocytes (107, 108). Later more studies were conducted on the immunosuppressive effects of EPIT. Mouse models of contact sensitivity (CS) (109, 110), experimental autoimmune encephalomyelitis (EAE) (111), collagen-induced arthritis (CIA) (112), and colitis (113) showed that, the epicutaneous application mainly exerted an inhibitory effect by inducing Ts/Treg cells, rather than simply suppressing Th1 type response or Th2 type immune response.

EPIT, involving transdermal administration of allergen under an occlusive dressing that promotes allergen absorption, was introduced as a treatment for allergies surprising early (114, 115), and has gradually been used to treat food allergies due to the increase prevalence. To date, EPIT’s researches on food allergy treatment mainly focus on the egg (ovalbumin, OVA), milk (116, 117), and peanut allergies. EPIT usually consists of the daily application of a new patch on designated skin locations for maintenance dosing, involving cutaneous exposure to micrograms of allergens. OIT protocols start with an initial dose-escalation phase and then the maintenance phase. However, unlike the OIT protocol, the patch’s allergen content remains constant during EPIT treatment, but the daily application time of the patch gradually increases. What’s more, the most commonly used product in research is a product called Viaskin^®^ (DBV Technologies, Bagneux, France). The Viaskin^®^ epidermal delivery system (EDS) forms an occluded chamber on the skin that generates moisture and releases allergen proteins from its support. The protein is then absorbed through the skin, where it interacts with epidermal immune cells (118). Researches show that the product has sound therapeutic effects in mouse models and patients with a peanut allergy aged 4-11. Another key fact to notice is that there are no reports of severe side effects in clinical studies, indicating high safety (118–121). Viaskin^®^ has now completed Phase III clinical trials (122) and is currently undergoing a five-year Open-Label Extension PEPITES study (PEOPLE) (123). The objectives who have now completed three years of active treatment in PEOPLE demonstrate that daily EPIT treatment for peanut allergy beyond one year leads to a continued response from a well-tolerated, simple-to-use regimen. Although Viaskin^®^ received the FDA’s Breakthrough Therapy Designation (BTD) in 2015, it is still under review and has not been approved for use or sale in any country/region. To be approved, like PALFORZIA, more clinical trials are needed, including larger cohort and more extended durations studies. It is necessary to obtain more data that can be used to evaluate and support the overall risk/benefit relationship related to the Biologics License Application (BLA), including the safety, efficacy, effective treatment dosage and expected treatment endpoints of Viaskin^®^ (124).

The skin is an active immune organ, in which the microbiome, chemical, physical and immune barriers form an interactive network that can prevent the invasion of foreign proteins and peptides and other macromolecules (125). Although this protective effect of the skin plays an important role in maintaining the body’s immune function, this protective effect will also limit the dose of allergens or drugs delivered to the skin during EPIT treatment, which greatly limits the therapeutic effect (83, 118). Therefore, the skin application of proteins and peptides may still be a challenge. For example, studies have shown that the delivery efficiency of Viaskin^®^ EDS is only about 10% (126), which may be the main reason for the poor therapeutic effect of this product. To improve drug or vaccine delivery, tape stripping (to remove of epidermis corneal layer) (127), the use of liposomes (128, 129), niosomes (130), and membranes equipped with microneedles (131) have been applied to increase the skin permeability.

In addition, in order to improve the efficiency of allergen delivery in EPIT treatment, Kumar et al. (132) applied a patch containing allergens and adjuvants [1,25-dihydroxyvitamin D_3_ (VD3) and CpG oligodeoxynucleotides (CpG-ODNs)] to the back skin of OVA-sensitized mice pretreated by ablation micro-fractional laser, which is called μEPIT here. The research results show that μEPIT can deliver 80% of the powder in the patch into the mouse within about 1 hour, a faster and more efficient EPIT treatment. It is also worth mentioning that CpG may be a good adjuvant of EPIT. As a monotherapy, an adjuvant or an ingredient of vaccines, animal experiments have proven its effect in infectious diseases, allergies, and oncological diseases (133). CpG can be administered by injection, inhalation, oral, or even vaginal routes, but the safety of various administration methods is still controversial. In the clinical trials of Peter et al. (134), a group of patients with hay fever showed that subcutaneous administration of allergen with CpG alleviates clinical symptoms in comparison with the placebo group. However, CpG injection often leads to many local and systemic adverse reactions, the intensity of which depends on the CpG dose (133). Local symptoms comprise pain, skin flushing, edema, and pruritus, moreover, systemic symptoms are more severe, which include headache, myalgia, fever, nausea, and vomiting (133). It is worth noting that compared with other routes, such as subcutaneous injection, epicutaneous administration of CpG seems to be safer and may not give side effects. Majewska-Szczepanik et al. (135) found that epicutaneous application of CpG with OVA antigen inhibits atopic dermatitis in mice. More interestingly, epicutaneously applied CpG was not absorbed and was not detectable in serum, indicating higher safety. The combination of CpG and ODN as an adjuvant has been confirmed to some extent, but determining a more effective combination of CpG may be one of the development directions of EPIT adjuvants in the future. Besides, to tailor this powder delivery technology for clinical uses, Wu’s team (136) developed a powder-laden dissolvable microneedle array (PLD-MNA) that can carry antigen powder for EPIT. Their research results confirmed that the PLD-MNA antigen presentation rate is close to 100%. This novel, safe, effective, and self-managed food allergy treatment method is expected to become a new food allergy EPIT method. In addition to destroying the skin barrier, Sayami et al. (137) also tried to improve the patch material to promote antigen presentation. They developed an allergen-containing hydrophilic gel (HG) patch to treat milk allergy. The protein layer formed on HG surface creates a concentration gradient that becomes the force driving protein penetration, thereby improving antigen delivery efficiency (138). More importantly, a milk-sensitized mouse model and clinical trial have confirmed this EPIT patch’s therapeutic effect on milk allergy (137).

## The Role of Treg Cells in EPIT

In 2011, Dioszeghy et al. (101) used Viaskin^®^ EDS loaded with OVA for EPIT treatment and analyzed the systemic cellular immune response of EPIT. They found that the percentage of CD25^+^Foxp3^+^ CD4 T cells in the spleen of EPIT-treated mice was significantly higher than those of the sham group. Later in the researches of Mondoulet et al. (139, 140) also showed that the mRNA expression of Foxp3 in the EPIT group was significantly higher than that of the control group. These studies all underlined the involvement of Treg cells in EPIT. In 2014, Dioszeghy et al. (141) further explored the role of Treg cells in EPIT. They first used Viaskin^®^ EDS loaded with peanut allergen to treat peanut-sensitized mice for eight weeks with or without anti-CD25 antibodies injection. Moreover, they found that EPIT significantly increased the proportion of CD4^+^CD25^+^Foxp3^+^ Treg cells in the spleen of peanut-sensitized mice. However, the proportion of CD4^+^CD25^+^Foxp3^+^ Treg cells in the spleen was lowered with the intraperitoneal injection of anti-CD25 antibody. Consequently, the EPIT treatment effect was inhibited at the system level, indicating the role of CD4^+^CD25^+^Foxp3^+^ Treg cells in the induction of immune tolerance by EPIT. In addition, they also transferred the CD4^+^CD25^+^ Treg cells induced by EPIT treatment to peanut-sensitive mice or Foxp3-IRES-mRFP mice, respectively, and determined the maintenance of Treg cells after EPIT termination and the ability to induce host Treg. In their study, both Foxp3^+^CD62L^+^ and Foxp3^+^CD62L^-^ Tregs increased significantly following EPIT. Yu et al. (136) used a PLD-MNA to treat mice with EPIT and analyzed CD4^+^ T cells in the spleen and LNs using flow cytometry. The results also confirmed that EPIT effectively induced CD4^+^CD25^+^Foxp3^+^ Treg cells in the spleen and LNs.

Moreover, to determine the Foxp3^+^ Treg cell subtypes that play a role in EPIT treatment, some studies have analyzed the expression of CD62L on its surface. CD62L is a marker utilized to distinguish naive cells from effector cells, is a crucial lymphoid homing molecule. After EPIT treatment of milk-allergic mice with Viaskin^®^ EDS containing milk, Mondoulet et al. (142) collected CD4^+^CD25^+^Foxp3^+^CD62L^+^/CD62L^-^ Treg cells from the mouse spleen. They transferred them to unsensitized mice before initiating peanuts. By measuring allergic indicators such as body temperature and mast cell protease-1 levels in mice in each group, it was found that compared with the positive control group, only mice in the CD4^+^CD25^+^Foxp3^+^CD62L^+^ Treg cells transfer group were protected from allergic reactions. This result indicated that CD4^+^CD25^+^Foxp3^+^CD62L^+^ Treg cells might play an essential role in the induction of immune tolerance through EPIT treatment. Furthermore, CD4^+^CD25^+^ Foxp3^+^CD62L^+^ Treg cells have been proved to be a Treg subtype with strong immunosuppressive effects that can prevent the occurrence of fatal acute graft-versus-host disease (GVHD) (143). Dioszeghy et al. (144) compared the phenotype and inhibitory activity of Treg cells induced by EPIT, OIT, and SCIT in peanut-sensitive mice. They found that a significant difference in the phenotype of EPIT-induced Treg cells was the induction of both effector/memory (Foxp3^+^CD44^hi^ CD62L^-^) Treg cells and naive (Foxp3^+^CD44^lo^CD62L^+^) Treg cells. In contrast, OIT and SLIT induced only effector/memory Treg cells. Moreover, whereas OIT- or SLIT-induced Treg cells lost their suppressive activity after discontinuing treatment, the suppressive activities of EPIT-induced Treg cells were still present at eight weeks after the end of treatment, suggesting that EPIT may induce a more long-lasting tolerance by inducing CD44^lo^CD62L^+^ naive cells. Another study monitored the changes in DNA methylation levels during the treatment of peanut-allergic mice with EPIT or OIT (142). Significant hypomethylation of the *FOXP3* promoter in mice was only observed in the CD62L^+^ Treg cells in the EPIT treatment group, which further verified the potential role of CD62L^+^ Treg cells in EPIT.

Except for Foxp3^+^ Treg cells, Foxp3^-^ Treg cells have also been confirmed to play a role in EPIT. Tordesillas et al. (145) show for the first time that the EPIT treatment with Viaskin^®^ EDS protected OVA-sensitized mice from anaphylaxis and supported the selective expansion of a population of unique gut-homing latency-associated peptide (LAP)^+^ Treg cells which can directly suppress mast cell activation and lead to sustained clinical protection. It was confirmed by using Viaskin^®^ EDS equipped with OVA-Alexa Fluor 647 in another research (146). Moreover, a high expression level of panmucosal homing marker CCR6 and gut-homing marker CCR9 were observed on the surface of these Foxp3^-^LAP^+^ Treg cells, showing that there was unique imprinting of gut-homing capacity on this Treg-cell subset (145). Furthermore, Dioszeghy et al. (147) found that EPIT treatment effectively induced the production of both CD25^+^Foxp3^+^CD62L^+^ Treg cells and Foxp3^-^LAP^+^ Treg cells in the LNs and spleen of mice. They also measured the Treg cell subtypes in the spleen and LNs after eight weeks of EPIT treatment and found that CD25^+^Foxp^+^CD62L^+^ Treg cells were still increased compared to two weeks of treatment, but no Foxp3^-^LAP^+^ Treg cells were observed (147). Dioszeghy’s research showed that the production of Foxp3^-^LAP^+^ Treg cells is temporary, and the author speculated that Foxp3^-^LAP^+^ Treg cells might participate in the first mechanistic steps of EPIT to induce CD25^+^Foxp3^+^ Tregs (147). Besides, we speculate that this may be related to the local effects of LAP^+^ Treg cells, and future studies should further examine the number and proportion of LAP^+^ Treg cells in the intestine.

The above research results indicate that EPIT may be a potentially safe, effective, and non-specific treatment for food allergies, which can induce Treg cells of a specific phenotype and immune tolerance. While EPIT could focus on the treatment of some severe food allergens, how the antigen induces the production of Treg cells remains unclear. Dioszeghy et al. (101) used flow cytometry to analyze the phenotype of immune cells in the skin and LNs after using viaskin^®^ EDS loaded with OVA on intact skin for different times. They found that when applied viaskin^®^ EDS on intact skin, the allergen is specifically captured by APCs, especially for DCs, and DCs would further migrate through the dermis to the LNs. Tordesillas et al. (146) applied Viaskin^®^ EDS loaded with OVA to mice’s skin to determine how antigen applied topically to healthy skin is acquired and presented by skin DC subsets to generate LAP^+^ Tregs. The results showed that CD11b^+^ CD64^+^ macrophages acquired most of the antigen reaching the dermis, and the OVA^+^ CD11c^+^ MHCII^+^ population in the dermis was predominantly CD11b^+^ cDC2 phenotype. However, only LCs and cDC2s were the main subtypes that presented antigens in the epidermis to the dLNs. Through further cell co-culture experiments (LC or cDC2s co-cultured with DO11.10 mouse CD4^+^ T cells) and animal experiments with anti-CSF1R (deplete LCs) or langerin-DTR mice or *CD11c-Cre x IRF4^fl/f^* mice, it was found that cDC2s, rather than LCs, are sufficient for the presentation of topical antigen to CD4^+^ T cells *in vivo*. Moreover, through co-culture with DO11.10 CD4^+^ T cells, they found that only PDL2^+^ cDC2s were able to induce proliferation of responder T cells and mainly promoted the production of LAP^+^CCR4^+^CCR6^low^ Treg cells. Dioszeghy et al. (147) also used Viaskin^®^ EDS loaded with OVA to study the mechanism by which EPIT treatment induces Treg cells and immune tolerance in an OVA-sensitized mouse model. The phenotypes of APCs and Treg cells in the skin and skin-draining LNs (sdLNs) were analyzed by flow cytometry. In agreement with Tordesillas et al. (146) findings, they found that the allergens in the skin of sensitized mice were taken up by LCs and cDC2s during EPIT treatment and migrated to the sdLNs to induce the production of both CD4^+^CD25^+^ Foxp3^+^CD62L^+^ Treg cells and Foxp3^-^LAP^+^Treg cells. However, Dioszeghy et al. (147) found that LCs depletion significantly reduced the migration of OVA^+^CD11^+^ cDC2s to sdLNs, and weakened allergens’ absorption and the induction of Foxp3^+^ Treg cells, especially Foxp3^+^CD62L^+^ Treg cells. These changes ultimately led to a failure to induce desensitization and sustained unresponsiveness (SU). The two research methods (146, 147) are basically similar, but Tordesillas et al. (146) did not pre-sensitize mice, so we speculate that the sensitization state of mice plays a vital role in the role of LCs in EPIT treatment. In Yu et al.’s research (136), they used intravital confocal imaging and flow cytometry to analyze the antigen uptake process after PDL-MNA loaded with OVA administration. The results showed that APCs’ main phenotypes that took up and processed the antigen in the skin were CD11b^+^CD11c^-^F4/80^+^ macrophage cells and CD11b^+^CD11c^+^F4/80^+^ macrophage-like cells, which is consistent with the findings of Tordesillas et al. (146). However, they did not find that DCs or LCs played a unique role in the antigen uptake process or conduct further analysis on the cells that play a role in antigen migration. In view of the difference between Viaskin^®^ EDS and PDL-MNA, we speculate that the integrity of the skin barrier may have an impact on the antigen presentation during EPIT treatment. In addition, they compared PLD-MNA with powdered allergens (EPIT) and liquid allergens (SCIT) for treatment. They found that powdered allergens are superior to liquids in attracting immune-regulatory macrophages and inducing immune tolerance in sensitive animals. Moreover, Tordesillas et al. (146) also tested antigen presentation and immune tolerance induction in hairless SKH1 mice with abnormal hair follicle development during EPIT treatment. They found that in SKH1 mice, the delivery of antigen to sdLNs was almost completely abolished, indicating that the integrity of hair follicles is also essential in the antigen presentation process of EPIT. More interestingly, this phenomenon is consistent with the observation that Treg cells in human skin are preferentially located in hair follicles, and that skin with high hair density has a higher proportion of Treg cells than skin with low hair density (148). Besides, Rodriguez et al. (149) also found that about 20% of CD4^+^ T cells in normal adult skin are Treg cells expressing specific surface molecules, and most of them have an activated effect memory phenotype, which provides conditions for EPIT to induce immune tolerance.

**Figure 2:**
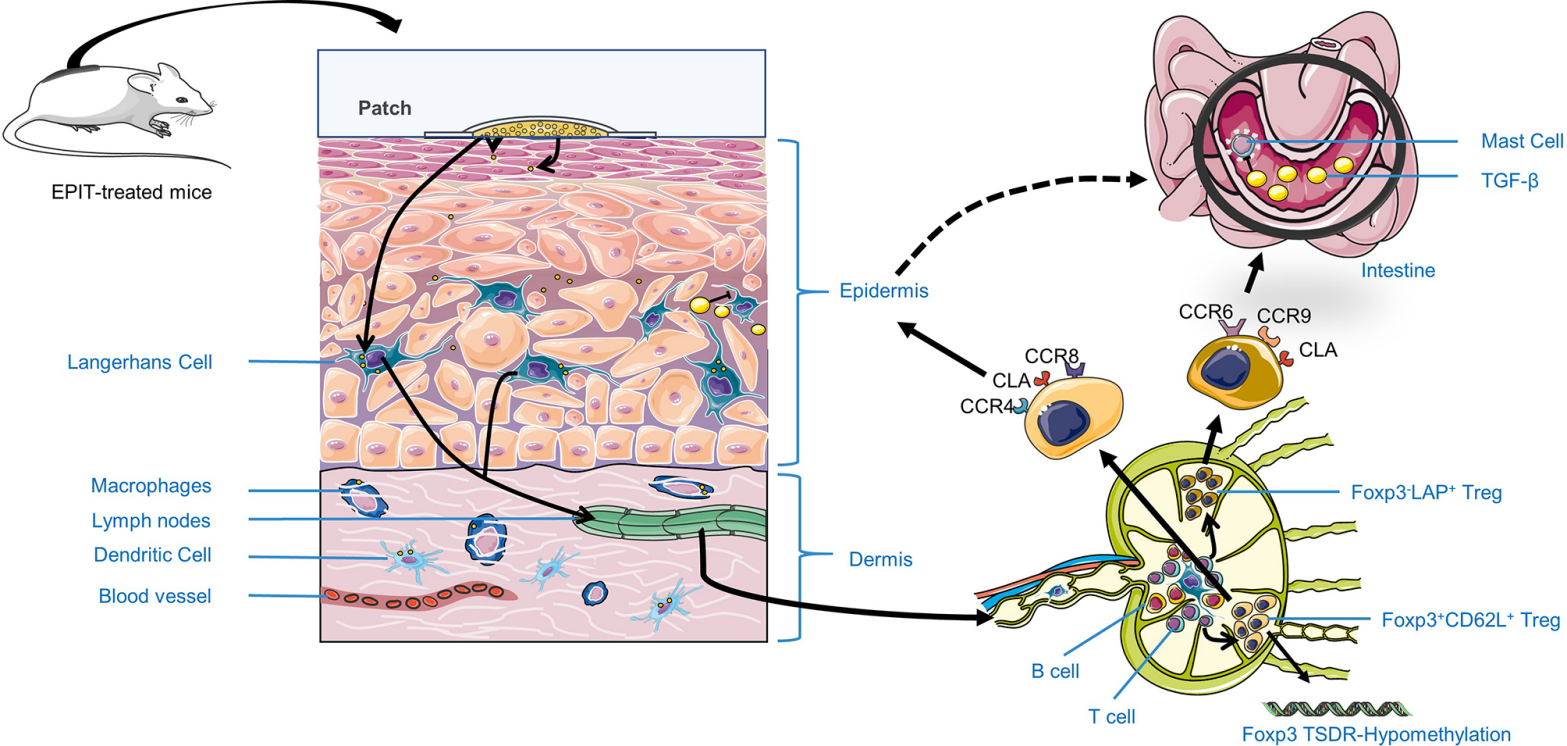
The possible mechanism of EPIT treatment of food allergy to induce immune tolerance. First, Antigen applied on the skin surface can be captured, processed, and presented in the LNs by Antigen-presenting cells (APCs), such as LCs in the epidermis, and macrophages, DCs in the dermis. Besides, they can promote more APCs aggregation by secreting TGF-β or IL-10. Second, APCs migrate to lymph nodes and promote naive T cells distinguish into Treg cells by secreting TGF-β, reducing Foxp3 TSDR methylation level or direct contact. Most importantly, EPIT will exert its immune tolerance by inducing CD4^+^CD25^+^Foxp3^+^ Treg cells of a specific phenotype, especially for Foxp3+CD62L^+^ Treg. Besides, Foxp3^-^LAP^+^ Treg may exert local effects by expressing intestinal homing molecules CCR6, CCR9, and skin-homing molecules CLA, CCR4, related to the higher safety EPIT treatment.

In summary, we can know that in EPIT, the antigens acting on the skin surface can be captured and processed by macrophages, DCs, and LCs, and then further presented by DCs and LCs in the LNs to naïve CD4^+^ T cells, thereby inducing immune tolerance. Nevertheless, which signaling pathways or signaling molecules involved are still being further explored. Dioszeghy et al. (144) found that the surface of CD4^+^CD25^+^Foxp3^+^Treg cells induced by EPIT expressed chemotactic cytokine receptors such as CXCR3 (Th1), CCR4 (Th2), CCR8 (Th2), CCR6 (Th17), CCR9 (gut), and CLA (skin), and the expression levels of CCR6, CCR8, CCR9, and CLA were maintained after the end of immunotherapy, suggesting the induction of a more long-lasting tolerance. Interestingly, only EPIT-induced CD4^+^CD25^+^Foxp3^+^Treg cells expressing CLA also expressed CCR9 (**Figure 2**), while OIT-induced Tregs expressed CCR9 but not CLA (144). The above results indicated that these CD4^+^CD25^+^Foxp3^+^ Treg cells induced through the skin have obtained intestinal homing properties, while in OIT treatment, Treg cells are locally induced in the mesenteric lymph nodes (mLNs) and only have gut homing properties. This may also be one of the reasons why EPIT rather than OIT can induce systemic immune tolerance. Tordesillas et al. (145) also showed that the use of EPIT to treat mice could produce specific LAP^+^Foxp3^-^ Treg subgroups that highly expressed CCR9 and CCR6. These Treg cells do not function by inhibiting IgE antibodies but directly inhibit mast cells’ activation, leading to sustained protection against food-induced allergic reactions. Furthermore, Dioszeghy et al. (144) also showed that EPIT-induced Treg cells are CTLA-4-mediated, rather than IL-10-dependent. More specifically, they used the *in vitro* restimulation of splenocytes in the presence of anti-IL-10 or anti-CTLA4 blocking antibodies to analyze the mechanisms of suppression by CD4^+^CD25^+^Foxp3^+^ Tregs cells. The suppression of Th2 cytokine production with EPIT was utterly blocked by anti-CTLA4 rather than anti-IL-10, indicating that the effect of EPIT on CD4^+^CD25^+^Foxp3^+^Treg cells is a CTLA-mediated action. Although IL-10 may not be involved in the induction of Treg cells, it still plays an essential role in EPIT treatment. In Yu et al.’s study (81), they found that the macrophage-like cells that produced TGF-β and IL-10 were significantly higher in the EPIT group treated with PLD-MNA than the SCIT group or the sham group. Moreover, the high levels of TGF-β and IL-10 in the skin of PLD-MNA-EPIT-treated mice seem to be consistent with the increased level of CD4^+^CD25^+^ Treg-like cells in the spleen, which indicates that IL-10 and TGF-β may play a role in EPIT treatment. However, its specific mechanism of action still needs to be further explored. Moreover, Mondoulet et al. (142) found that EPIT can lead to a unique and stable epigenetic signature in specific T cells, namely the Th2 cell *Gata3* promoter hypermethylation and Foxp3^+^CD62L^+^ Treg cell *Foxp3* promoter hypomethylation. This specific epigenetic signature is compartments with the down-regulating key Th2 regulators and up-regulating Treg transcription factors, which may explain the sustainability of protection and the observed bystander effect.

As far as current research is concerned, the mechanism of EPIT for food allergies can be summarized as follows (**Figure 2**). First, Antigen applied on the skin surface can be captured, processed, and presented in the LNs by Antigen-presenting cells (APCs), such as LCs in the epidermis, and macrophages, DCs in the dermis. Besides, they can promote more APCs aggregation by secreting TGF-β or IL-10. Second, LCs and cDC2s migrate to the LNs, and cDC2s promote T cells by secreting TGF-β, directly contacting (as described in the regulatory T cell section), reducing *Foxp3* TSDR methylation levels or upregulating GATA3 methylation levels (142) to down-regulate Th2-type immune response. In addition, EPIT exerts its immune tolerance by inducing Treg cells of a specific phenotype, especially CD4^+^CD25^+^Foxp3^+^CD62L^+^ Treg cells, which play an essential role in the induction of immune tolerance. Simultaneously, Foxp3^-^LAP^+^ Treg cells may be involved in the generation of CD4^+^CD25^+^Foxp3^+^ Treg cells and act locally by expressing chemotactic cytokine receptors as CCR6 and CCR9, which are related to the induction of systemic immune tolerance in EPIT treatment (**Figure 2**). Finally, EPIT-induced Treg cells may inhibit the effects of B cells, mast cells, and eosinophils through the direct mechanism (1–3), and (7) described in the regulatory T cells section. This hypothesis is supported by EPIT’s observed effectiveness in these allergic mice [the reduction of clinical symptom scores, decrease in allergen-specific IgE (sIgE) levels, increase in IgG levels, and inhibition of mast cells and basophils’ infiltration]. The specific mechanism of action of EPIT-induced Treg cells to induce immune tolerance remains to be further confirmed.

## Perspectives

**Figure 3:**
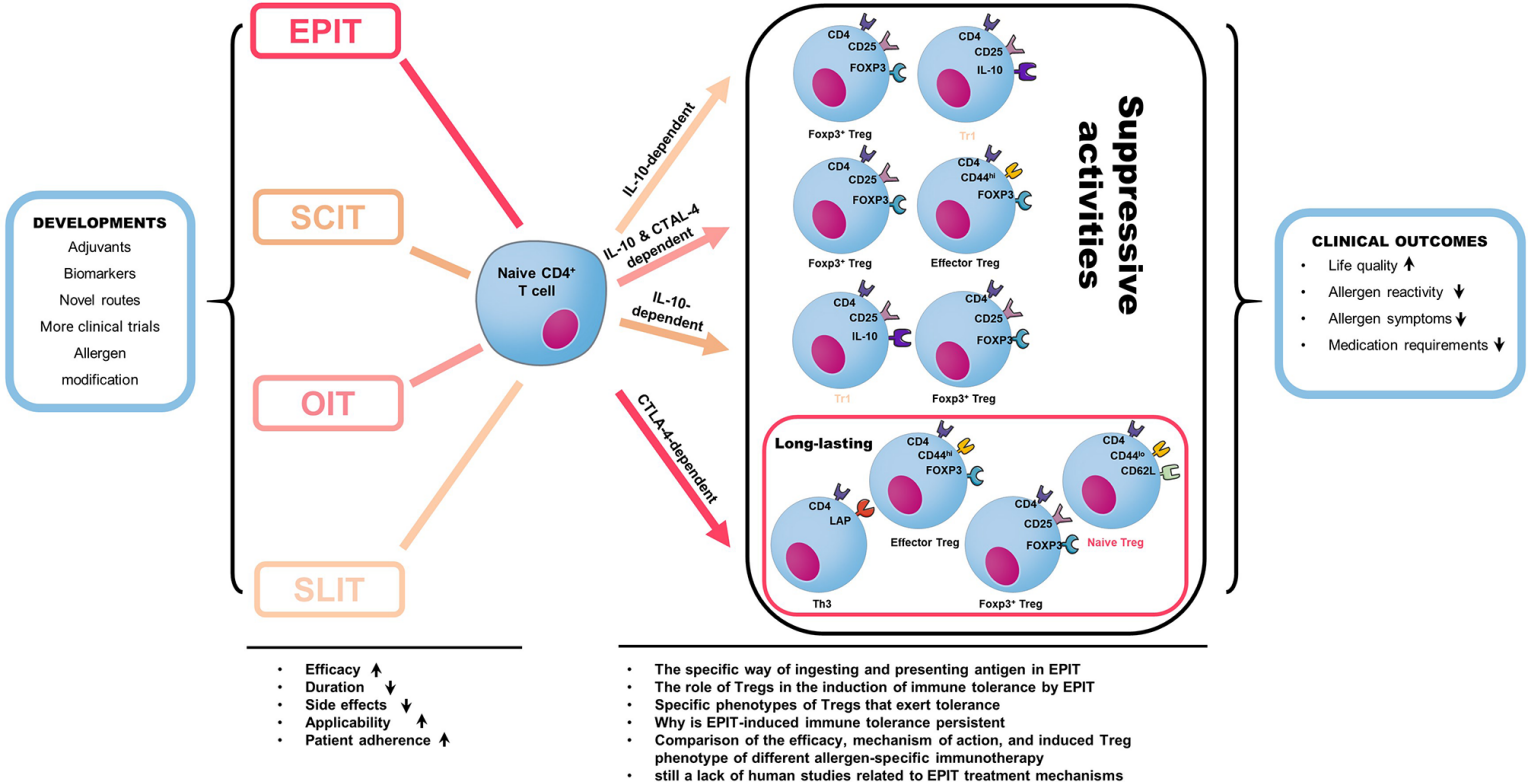
Different allergen-specific immunotherapy can induce the production of different regulatory T cell subtypes. CD4^+^CD25^+^Foxp3^+^ Tregs were induced by the three treatment routes but with more significant numbers induced by EPIT. This difference is due to an increase in naive Tregs in EPIT because the induction of effector Tregs was similar in EPIT, OIT, and SLIT, and only EPIT induced naive Tregs. EPIT and OIT also increased the level of CD4^+^LAP^+^ cells (Th3), whereas SLIT induced IL-10^+^ Tr1 cells. The suppressive activity of EPIT-induced Tregs did not depend on IL-10 but required CTLA-4, whereas OIT acted through both mechanisms, and SLIT was strictly dependent on IL-10. Furthermore, whereas OIT- or SLIT-induced Tregs lost their suppressive activities after treatment was discontinued, the suppressive activities of EPIT-induced Tregs were still effective eight weeks after the end of treatment, suggesting the induction of a more long-lasting tolerance. Moreover, Allergen-specific immunotherapy is the only option for the long-term cure of allergic diseases. It has been used for food allergy treatment research in recent years. The latest developments in the specific field of allergy and immunology aim to improve the efficacy, applicability, and patient compliance with treatment while reducing side effects and duration. Novel administration routes, the definition of biomarkers for better monitorization of therapy success, novel adjuvants for increased AIT efficacy, and the development of allergen preparations with increased antigenicity and decreased allergenicity allergoids all serve these crucial goals. The final result of AIT is called allergen-specific tolerance, which is a state of active immune response due to changes in immune cell function. A successful AIT’s clinical outcome can reduce drug demand, allergen reactivity, and allergic symptoms and improve life quality.

The current mouse model provides essential insights into the EPIT mechanism. However, the mechanism of immune tolerance induced by EPIT has not yet been fully explained. First, we need to determine the specific types of APCs that play a role in EPIT therapy. For example, Dioszeghy et al. (147) reported that LCs are required to induce Treg cells. In contrast, Tordesillas et al. (146) suggest that LCs are redundant and CD11b^+^ cDC2s are sufficient to present topical antigen to CD4^+^ T cells *in vivo*. Whether the sensitization status of mice or other mechanisms plays a crucial role in this difference remains explored. Besides, the effects of other APCs, including macrophages, need to be further studied. In addition to specific APCs, Toshiyuki et al. (150) confirmed the contribution of Notch signaling to the establishment of sustained unresponsiveness to food allergens by OIT. The cytokines and signaling pathways involved in the process of antigen presentation by EPIT also required further study. Once these processes are transparent, adjuvants could be used to target APCs and other molecules to assist AIT treatment in promoting the efficacy or reducing the side effects of the treatment. For example, Korotchenko et al. (151) used carbohydrates coupled with allergens to target and stimulate DCs. It shows that the IgE-binding ability of the new glycoconjugate could be reduced, and the side effects of EPIT treatment were significantly reduced. Second, it is necessary to explore further the type, phenotype, and function of Treg cells produced by EPIT, such as Foxp3^-^LAP^+^ Treg cells and CD4^+^CD25^+^ Foxp3^+^CD62L^+^ Treg cells. Determining which type of cells exerts immune tolerance will provide new insights for Treg cells as a new type of immunotherapy target to treat food allergies. Third, it is necessary to explain how EPIT therapy induces systemic immune tolerance, including how it alleviates skin, digestive, and respiratory allergies and, more importantly, clarifies the role of Treg cells. Fourth, we should also compare Treg cells’ role in different allergen-specific treatments, especially the phenotype, function, and persistence of the Treg cells produced by different methods. Finally, there is still a lack of human studies related to EPIT treatment mechanisms, which can provide an essential theoretical basis for EPIT treatment effectiveness and future practical applications (**Figure 3**).

A better understanding of allergen tolerance’s underlying mechanisms and the roles and interactions of cells will support developing a more suitable, easily administered, durable, effective, safe, and patient-friendly treatment. EPIT, as a potential treatment for food allergies, has been shown to have high safety and specific therapeutic effects. Because the efficacy of EPIT is still limited, elucidating its mechanism of action and improving its efficacy is still the focus of current research. Improving the allergens used in EPIT treatment, and the development of adjuvants are potential research directions to enhance the efficacy of EPIT. EPIT may be a genuinely effective new method for treating food allergies, but it is clear that our work is not yet done, and the best treatment protocol and mechanisms need to be elucidated.

## Author Contributions

GL mainly completed the writing and sorting of the article. ML, JW, and YM provided some ideas and insights. HC revised the article. All authors contributed to the article and approved the submitted version.

## Funding

This research was supported by National Natural Science Foundation of China under grant No. 81773435.

## Conflict of Interest

The authors declare that the research was conducted in the absence of any commercial or financial relationships that could be construed as a potential conflict of interest.
